# Polyunsaturated Fatty Acids Mediated Regulation of Membrane Biochemistry and Tumor Cell Membrane Integrity

**DOI:** 10.3390/membranes11070479

**Published:** 2021-06-28

**Authors:** Souvik Mukerjee, Abdulaziz S. Saeedan, Mohd. Nazam Ansari, Manjari Singh

**Affiliations:** 1 Department of Pharmaceutical Sciences, Guru Ghasidas Vishwavidyalaya (A Central University), Bilaspur 495009, Chhattisgarh, India; mukherjees388@gmail.com; 2 Department of Pharmacology & Toxicology, College of Pharmacy, Prince Sattam Bin Abdulaziz University, Al-Kharj 11942, Saudi Arabia; a.binsaeedan@psau.edu.sa; 3 Department of Pharmaceutical Sciences, Assam University, Silchar 788011, Assam, India

**Keywords:** biological membrane, breast cancer, polyunsaturated fatty acid, membrane remodeling, P–L–P membrane channel, ALA and GLA

## Abstract

Particular dramatic macromolecule proteins are responsible for various cellular events in our body system. Lipids have recently recognized a lot more attention of scientists for understanding the relationship between lipid and cellular function and human health However, a biological membrane is formed with a lipid bilayer, which is called a P–L–P design. Our body system is balanced through various communicative signaling pathways derived from biological membrane proteins and lipids. In the case of any fatal disease such as cancer, the biological membrane compositions are altered. To repair the biological membrane composition and prevent cancer, dietary fatty acids, such as omega-3 polyunsaturated fatty acids, are essential in human health but are not directly synthesized in our body system. In this review, we will discuss the alteration of the biological membrane composition in breast cancer. We will highlight the role of dietary fatty acids in altering cellular composition in the P–L–P bilayer. We will also address the importance of omega-3 polyunsaturated fatty acids to regulate the membrane fluidity of cancer cells.

## 1. Introduction

A fatal disease that originated in 460–370 BC is slowly engulfing modern civilization, a disease that scientists have termed cancer. From a chemical point of view, just as matter is formed by the combination of atoms, our body organ system is developed according to this parasitic trend from cells to tissues, from tissues to organs, and from organs to body systems [1]. Each cell protects its existence through division. After a certain period, each cell dies. Normal cell division becomes defective due to a stimulus or an external infection. When the cell divides at a much higher rate than the normal division rate, it is called a tumor [2]. If the cell spreads in the body system and then forms a new tumor, then the division of these cells is uncontrolled, and the cells gain immortality; then, it is termed cancer. More than 100 cancers have been discovered so far. For many years, scientists have been working tirelessly to bring this disease under control [3].

At present, scientists have rediscovered the importance of cell membranes to deliver different chemotherapeutic agents in cancer. This is because the cell membrane is a thin membrane that protects the cell from external injury and protects the cell organ within the cytoplasm. This cell membrane comprises three layers of protein–lipid–protein and 52% protein, 40% lipid, and 8% carbohydrate [4,5,6,7,8]. In cancer, a large amount of fatty acid biosynthesis occurs in the cell membrane, resulting in new membranes in tandem with uncontrolled cell division [9]. In the case of breast cancer, not only are fatty acids and phospholipids converted into a cell membrane, but the membrane’s biophysical atmosphere is also affected. Breast cancer is on the rise among women all over the world [10]. This disease is the most common after skin cancer. In this type of cancer, the estrogen receptor driven by the estrogen hormone in the cell membrane plays a crucial role. Cell membrane composition and its function are being routinely tested to restore normalcy and prevent cancer [11,12,13]. The role of omega-3 polyunsaturated fatty acids (PUFAs) in controlling tumor cell membrane fluidity, drug resistance, and altered membrane biophysics are still under question.

On the other hand, it has been found that saturated fatty acids increase the risk of breast cancer [14,15]. Omega-3 PUFAs are not directly synthesized in our body, and they must be taken from outside. All these fatty acids are found in various animal and vegetable sources [16,17,18,19]. This review will discuss how the cell membrane changes in estrogen receptor-positive (ER+) breast cancer. Why have polyunsaturated fatty acids been chosen instead of saturated fatty acids? How do these types of fatty acids prevent breast cancer by altering membrane biophysics?

## 2. Biophysical Architecture of Cellular Membrane

In the nineteenth century, a scientist named Charles E. Overton discovered that the membrane that protects cells from external injuries is lipid in nature. Lipids are one of the cell membrane building blocks covering all the cells in a cell’s cytoplasm [20]. Finally, in 1972, after conducting various experiments, two scientists, Sanger and Nicolson, described the cell membrane as looking a lot like a fluid mosaic. Thereafter, the cell membrane was named as fluid mosaic model. The cell membrane is composed of a lipid distillation in which some proteins are immersed [21]. This fluid mosaic model that renovated by Sanger and Nicolson is universally accepted till date [22]. Communication between one cell and another is carried out through many receptors and signal transduction pathways [23]. It also helps in the activities of different types of enzymes, including fusion, fission, endocytosis, and the exchange of various substances [24].

This lipid bilayer is responsible for the normal formation of each cell membrane. It can be easily seen using an electron microscope. The animal cell membrane is made up of lipid molecules and is around 50% of its mass [25]. The 1 × 1 µm space of the two-layer lipid contains approximately 5 × 10^6^ lipid molecules. Again, the cell membranes of small animals contain (approximately) 10^9^ lipid molecules. There are various types of membranes present in eukaryotic cells, such as plasma, endosomal, nuclear, and mitochondrial membranes, where lipid composition is specified in each membrane. Lipid composition is mainly cholesterol/phospholipid and is also influenced by lipid raft formation [26]. In animals and bacteria, the tails of cell membranes are usually made up of fatty acids, they can differ in length of carbon chains (usually exists between 14 and 24 carbon atoms). A tail usually has one or more cis doubles, i.e., unsaturated, and another tail is saturated. A small kink in the tail is created by each double bond. This creates a difference in the length and fullness of the fatty acid [27]. This difference affects how the phospholipid molecules entangle each other and affect the fluidity of the cell membrane. Phosphoglycerides are the main component of biological membranes that have a three-carbon glycerol backbone and two long-chain fatty acids attached to the adjacent carbon atom of the glycerol molecule through an esteric bond. The two fatty acid chains attached to the molecule of glycerol are non polar hence hydrophobic while the polar heads which mainly consists of the phosphate group attached to the third carbon of the glycerol molecule is hydrophilic. Many fatty acids and head groups combine to form different phosphoglycerides in the cell membrane [28,29,30,31].

**Figure 1:**
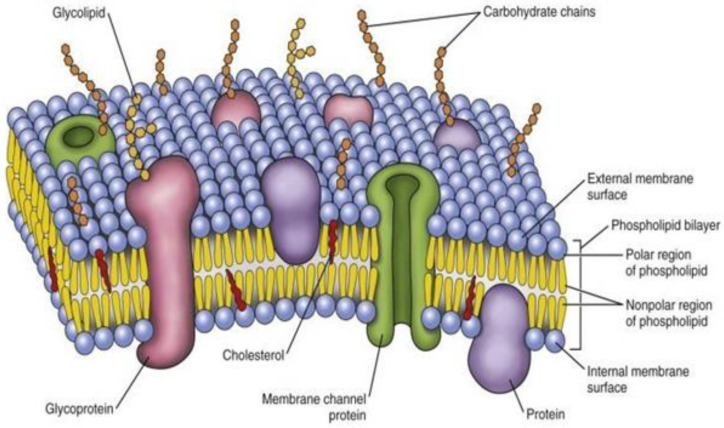
Components and layers of biological membrane.

Another essential phospholipid is sphingomyelin, which is made from sphingosine. Sphingosine is a long acyl chain, having one amino group and two hydroxyl groups. In this sphingomyelin, the fatty acid tail is attached to the amino group, and the phosphocholine group is attached to the terminal hydroxyl group. This free hydroxyl group polarizes the edges of this phospholipid because it can quickly form a hydrogen bond. In this way, phosphatidylcholine, phosphatidylserine, phosphatidylethanolamine, and sphingomyelin interact with the lipid water molecules and create more than half of the mass [32]. In addition to all these phospholipids, there are also two layers of cell membranes in the lipid layer, namely cholesterol, which is present in a large amount, and glycolipids. There is cholesterol for each phospholipid molecule. Cholesterol is a type of sterol made up of a single polar hydroxyl group and a non-polar hydrocarbon chain (Figure 1). Now, we will discuss how differential lipid is synthesized and controlled in the cell membrane [33,34,35].

### Biosynthesis of Fatty Acids in the Cell Membrane

**Figure 2:**
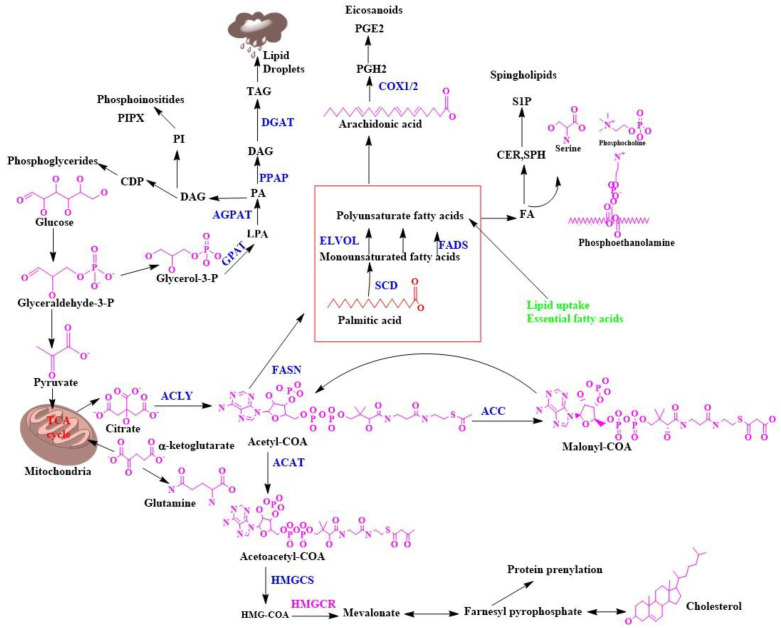
Biosynthesis of lipids in biological membranes.

Lipid metabolism in eukaryotic cells is accomplished by forming various chemical compounds centered on many metabolic reactions. The lipid is mainly divided into six parts, including the following: (1) Fatty acid mainly acts as a stimulant of lipid biosynthesis and produces metabolites containing oxygen. (2) Free sterols act as a cell membrane shape provider. (3) Sterol esters are mainly made from fatty acids and sterols. (4) Triacylglycerol is made from glycerol and fatty acid. (5) Phospholipids are mainly made from fatty acids, glycerol, and alcohol. For example, inositol, serine, choline, and ethanolamine act as a cell membrane shape provider [36]. One of the major phospholipids in the biological membrane is phosphoglyceride, which is an important substrate for various lipid kinases and phosphatases. As a result, it can form various biologically active compounds, which are called phosphoinositides. They are acidic phospholipids present in the cell membrane and interact with each membrane compartment protein and are phosphorylated with the help of phosphoinositide kinases [37]. (6) Sphingolipids are long fatty acids and are responsible for cell surface formation. As a result, endocytosis, cell cycle control, etc., are efficiently completed. The so-called lipid class discussed above is made up of acetyl COA in the cell’s cytoplasm. Lipid biosynthesis mainly requires two-branched acetyl COA, one part of which produces sterol and the other part fatty acids, which paves the way for triacylglycerol biosynthesis [38,39,40,41,42,43]. In eukaryotic cells, there are several types of membranes present, such as plasma, endosomal, nuclear, and mitochondrial membranes. Specific lipids are present in certain levels within a particular membrane and help the cell membrane move in a particular way [44]. An essential component of this eukaryotic cell is cholesterol, which regulates the cell membrane’s speed and function (Figure 2). It is the last component of the sterol biosynthetic pathway, and this component forms a semipermeable barrier that regulates membrane fluidity [45].

## 3. Membrane Biophysical Difference between a Normal Cell and Cancer Cell

**Figure 3:**
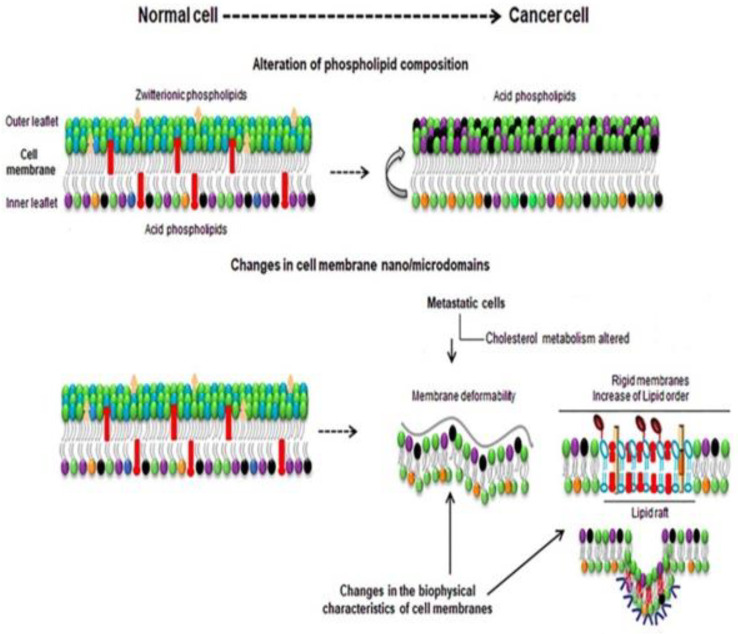
Composition and biophysical changes of a normal cell and cancer cell.

We know that when a tumor cell turns into a malignant cell, that cell spreads to every part of the body very quickly with the birth of new malignant cells, resulting in the formation of a new cell membrane in each cell. Important components vary in the path of lipid biosynthesis (Figure 3) [46]. This lipid synthesis gradually decreases and increases in the cancer cell and creates new cell membranes, depending on the nature, stage, etc., of different cancer cells, and depending on the other lipids in the cell membrane. We discussed in the last portion that lipid molecules are formed by the connection of the polar head and hydrophobic tail. A thermodynamic force is mainly responsible for the shape of the lipid molecule. This force helps the cell membrane to form micelles or bi-layered sheets [47]. Due to the presence of lipid polar head groups, differences in shape, charge, character, etc., are seen in the membrane, such that the long polar group phosphatidylcholine, due to the amphiphilicity of sphingomyelin, looks much like a cylindrical shape. Sphingolipids have long fatty acid chains and are converted from solid to gel by sterol. Asymmetry of lipid can be seen inside and outside the cell membrane of a normal cell. Phosphatidylcholine and sphingomyelins are zwitterionic lipids located outside of the cell membrane.

On the other hand, phosphatidylethanolamine is a phospholipid that is comprised of glycerol esterified with two fatty acids and phosphoric acid where the phosphate group is attached with choline and forms phosphatidylcholine. Phosphatidylcholine is combined with ethanolamine and forms phosphatidylethanolamine. They are located in the inner leaflet of the cell membrane. The presence of phosphoethanolamine in the inner leaflet creates a negative charge on the cell membrane surface. It facilitates the passage of various hydrolysis processes such as phospholipase C to inositol 1,4,5-triphosphates, and diacylglycerol that act as secondary messengers [48].These substances are again called a kind of secondary messenger. When a normal cell turns into a cancer cell, all the changes mentioned above can be noticed; for example, a negative charge is created outside of the cell membrane in the cancer cell, as a result of which the environment outside the cell membrane becomes acidic and the fluidity of the membrane changes [49]. The different shapes of the lipids in the cell membrane, depending on the temperature, change from lipid gel to solid, increasing their hydrophobic parts, but there is no change in the hydrocarbon tail. The fluidity of a cell membrane depends on the cholesterol in it. When the cell membrane changes to a liquid state, it contains 8–15% cholesterol. Depending on the nature of cancer, cholesterol metabolism repeatedly changes in the cell membrane [48]. In metastatic cells, cholesterol levels are lower than in normal cells. In the case of multidrug-resistant cells, cholesterol levels are high; thus, the membrane becomes more rigid and less permeable for drugs, developing drug resistance. In addition to cholesterol, any other above-discussed molecules are higher in multidrug-resistant cells than normal cells. Increasing cholesterol levels increases the malignant transformations, hyper-growth, and invasiveness. Lipid rafts also interact with other proteins such as integrins, CD44, and CD24. These receptors are involved in tumor progression [50,51,52].

## 4. Pivotal Role of Membrane Lipid and Cholesterol for Reprogramming of Breast Cancer

Breast malignant growth cells often show explicit changes in their metabolic movements. The most popular metabolic irregularity connected to disease cell science is the Warburg impact, which expands glycolytic transition, diminishes TCA cycle motion, and expands glutamine usage for anabolic pathways [53,54,55,56]. Metabolic reconstructing through a change in lipid biosynthesis and cholesterol blend is additionally a sign of breast cancer but is poorly understood. This metabolic reconstructing upholds the expanded creation of metabolic intermediates for the union of proteins, nucleic acids, and lipids. All these are essential for the fast multiplication of breast cancer cells. It has been demonstrated that mutations in major oncogenes, that PI3K/AKT, KRAS, and MYC, mediate metabolic shifts in cancer cells and activate de novo fatty acid(FA) synthesis [57,58]. Profoundly proliferative breast malignancy cells fulfill the need for lipid/cholesterol by either expanding the taking up of exogenous lipids and lipoproteins or persistently reprogramming their endogenous biosynthetic pathways. FA biosynthesis is a contributory element of early phase malignancy advancement, disease cell development, and endurance. Unnecessary lipids and cholesterol in disease cells are stored in lipid drops. This is critical because raised lipid beads and stored cholesteryl ester content in tumors are viewed as signs of disease forcefulness [59,60,61,62,63]. These lipids arise from acetyl Co A and contain FA. The FA building blocks are obtained from either exogenous sources or amalgamation of FA. In this manner, it is essential to see how lipid/cholesterol biosynthesis is upgraded in malignant growth, because most normal human cells favor exogenous sources. However, tumors blend FA again and regularly show a shift toward FA combination [64]. From a robotic viewpoint, it is intriguing to take note that, by and large, the endogenous combination of lipids transfers physiological/pathophysiological prompts that synthetically indistinguishable exogenous lipids cannot mirror. Oncogene-changed disease cell requirements increase the amount of cholesterol to help their rapid development [65].

Notwithstanding upgraded cholesterol amalgamation again, the interaction of cholesterol take-up is likewise firmly directed by the flagging movement of the epidermal development factor receptor (EGFR), which is expanded in malignant growth. Now, we discuss how omega-3 PUFAs mediate regulation of membrane channel and tumor cell membrane fluidity [66].

## 5. Introductory Concept of Omega-3 PUFAs

Omega-3 PUFAs gather fundamental polyunsaturated unsaturated fats that assume significant roles in biological cell construction and cell flagging. Assignment 3 or 6 underlies this mechanism, alluding to the two-fold bond on the third or sixth carbon separately from the methyl branch. The most plentiful dietary polyunsaturated fatty acids are the short-chain omega-3 alpha-linolenic acids (ALAs), which are obtained from plant oils [67,68]. The longer chain omega-3 PUFAs, eicosapentanoic acid (EPA), and docosahexaenoic acid (DHA), usually referred to as marine unsaturated fats, are most proficiently obtained from fatty cold water fish such as salmon. They should be obtained from ALA and linolenic acid (LA) separately [69]. The desaturases and elongases have a more special proclivity for ALA than LA due to the overall 10-fold higher admission of LA, and, for the most part, more AA than EPA and DHA is formed. Whether ingested or synthesized, PUFAs are either oxidized for fuel, stored in triacylglycerol, taken up in phospholipid films for inevitable use as substrates by cyclooxygenase (COX) and lipoxygenase (LOX) compounds, or utilized as ligands for G receptors [70,71,72]. Either LA or ALA is changed into bioactive lipids due to low uptake into phospholipid films. In any case, 5 to 10% of both LA and ALA can be changed into the longer chain PUFAs, which are promptly taken up in phospholipid layers and structure the substrates for transformation to bioactive lipid items COX and LOX proteins [73]. The omega-3 unsaturated fats EPA and DHA and their subordinates are significant for retinal and mental health, intellectual capacity, and the creation of negligibly reactive eicosanoids, proresolving mediators named resolvins, and different tissue protectins [74].

**Figure 4:**
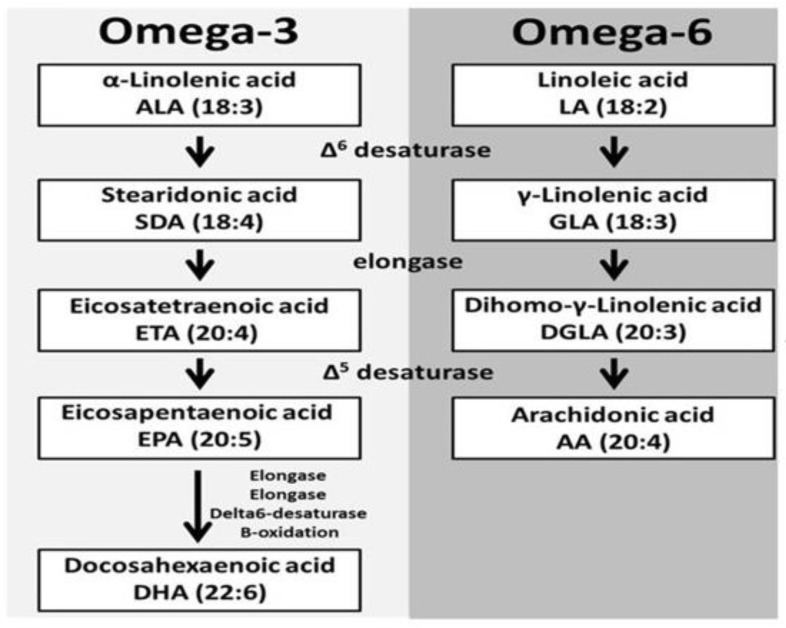
Polyunsaturated fatty acid biosynthesis.

However, most of the bioactive lipid mediators of interest are a consequence of COX and LOX compound movement in the long-chain PUFAs. EPA, DHA, AA, and 15-LOX follow up on the short-chain LA to shape 13(S)-hydroxyoctadecadienoic. Cancer is likely known to increase mammary tumor expansion. EPA and DHA contend with AA as substrates for COX and LOX proteins, even though EPA is a less suitable substrate than AA, especially for COX. Upon inflammatory stimulus, the catalyst phospholipase A2 discharges AA from phospholipid layers of monocytes, and overwhelmingly proinflammatory subsidiaries are created. COX-1 and COX-2 chemicals are liable for AA-inferred prostaglandin E2 and another arrangement of two prostaglandins and thromboxanes. 5-LOX, 12-LOX, and 15-LOX are responsible for the arrangement of four leukotrienes and lipoxins (Figure 4) [75,76].

## 6. Intermediate Cross-Talk of ALA and Gamma-Linolenic Acid (GLA)

The more significant part of the work evaluating how ALA and GLA may diminish breast malignancy hazard has been studied through in vitro or transgenic mouse models. A decrease in oncogenic protein is monitored through disturbance of plasma film lipid rafts, a decrease in cytokine production, and an expansion in apoptosis following the enactment of the plasma layer GRP120 protein [77,78,79,80]. ALA and GLA disturb lipid rafts, sphingolipid/cholesterol-improved miniature spaces of plasma layers that streamline motioning by concentrating proteins. Lipid rafts are especially significant for a few tyrosine kinase receptors. Decreases in epidermal development factor receptor and human epidermal development factor-2 receptor level and enactment have been exhibited in changed and harmful cells. A lessening in epidermal development factor receptor and human epidermal development factor-2 flagging would be relied upon to diminish multiplication [81]. A reduction in Ki-67 has been observed in favorable and dangerous mammary tissue after ALA and GLA supplementation in most preclinical models. Other preclinical studies highlight that ALA and GLA increase expression of BRCA1/2, phosphatase, and tensin homolog (PTEN) and different proteins related to the cell cycle and DNA repair [82,83,84].

## 7. Mechanism of GLA for Breast Cancer Prevention

The upstream omega-6 GLA may possess anti-cancer effects especially breast cancer. and it is a guaranteed dietary hotspot for disease counteraction and treatment. Nonetheless, the upstream omega-6 can be viably changed into arachidonic acid (AA) by a progression of unsaturated fat digestion chemicals. Upon uptake, LA (the antecedent of omega-6) will be changed into GLA within sight of ∆-6 desaturase (D6D), trailed by a two-carbon bond stretching by elongase to become DGLA, and lastly will be de-immersed by ∆-5 desaturase (D5D) to shape AA. COX is a major lipid peroxidizing enzyme, omega-6s can undergo a free radical pathway during lipid peroxidation and produce different PUFA-inferred metabolites [85,86]. For example, GLA and AA, both significant substrates for COX, can create single-arrangement prostaglandins (PGs-1) and double-arrangement prostaglandins (PGs-2) separately during COX-catalyzed lipid peroxidation. DGLA and AA can utilize various free radical pathways during lipid peroxidation and produce unmistakable free radical metabolites [87]. GLA may apply the opposite disease impact by creating prostaglandin E1 (PGE1) and the restrictive free radical metabolites from its COX-catalyzed lipid.

However, all the omega-6 can be directly consumed in the everyday diet. LA, the forerunner of omega-6, is more bountiful in plant seeds and oils and consequently is viewed as the fundamental dietary wellspring of all omega-6. Studies show that LA can be involved in both pro and hostile to malignant activities. For instance, LA invigorates cell expansion in the human bosom. LA can be desaturated and changed into GLA, which is catalyzed by the D6D protein. GLA has appeared to apply the opposite disease expansion impacts by affecting quality and protein articulation, disturbing cell cycle movement and inciting apoptosis. GLA from basic primary segments of the cell, atomic, and organelle layers directly affects ordinary cell capacity and digestion [88]. GLA is joined to the phospholipid film layer and is induces cell film cooperation—for example, cell–cell grip and receptor flagging framework. GLA sets off cytochrome c delivery related to changes in mitochondrial digestion and expanded caspase 3 movement, accordingly prompting cell apoptosis [89]. GLA modifies mitochondrial digestion and construction by affecting mitochondrial film arrangement and diminishing hexokinase and carnitine palmitoyltransferase I activity, accordingly, and promoting apoptosis. The primary impacts of GLA in ER+ breast malignancy intercede through the ER pathway [90].

## 8. Role of Omega-3 PUFAs in the Regulation of Membrane Channel Activity

**Figure 5:**
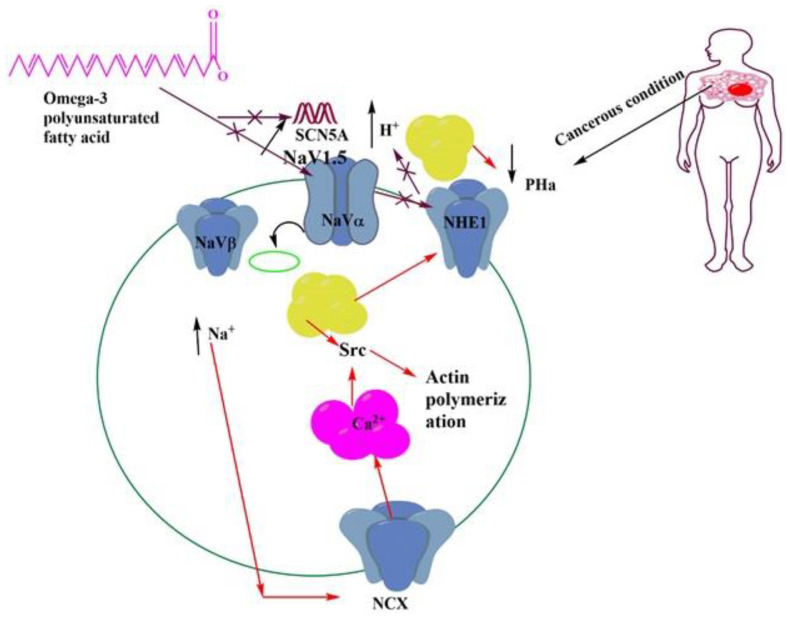
Omega-3 PUFAs in the regulation of channel activity and prevention of cancer progression.

Various experiments have shown that omega-3 PUFAs exhibit anti-invasive and antimetastatic properties by interfering with various functions of ion channels present in the biological membrane. However, we discuss the role of ion channels in the biological membrane for the spread of cancer and we also discuss how omega-3 PUFAs help in cancer prevention by targeting the ion channel [91]. The presence of approximately 300 types of ion channels can be observed in the cell membrane—for example, voltage-gated, ligand-gated, and lipid-gated ion channels, etc. However, various studies have shown that the voltage-gated sodium (Na_v_) channel plays an important role in cancer in all these ion channels [92]. The ions enter the cell membrane through the transmembrane of the semipermeable cell membrane. This Na_v_ channel is a transmembrane protein that is activated by the electrical charge created in the cell membrane and helps the sodium ion to travel through the membrane. Electrical potential in the membrane helps to open and close this channel [93]. This channel is formed by the insertion of a single polypeptide chain and four homologous domains through alpha and beta subunits covered by protein. Each subunit works differently in the membrane. When the protein of the alpha subunit is secreted, it enters the channel through sodium voltage, while the protein of the beta subunit remains in the visible state. The alpha subunit has four domains with six membrane-spanning segments in each: s1 to s6. This alpha subunit ion uses pores and forms one or two subunits [94]. The protein of this alpha subunit is mainly divided into nine parts that are represented by the Na_v_ signal, e.g., Na_v_1.1 to Na_v_1.9. The protein of each alpha subunit is differentiated by gene sequence, such as SCN1A for Nav 1.1, SCN2A for Nav1.2, SCN3A for Nav1.3, SCN4A for Nav1.4, SCN5A for Nav1.5, SCN8A for Nav1.6, SCN9A for Nav1.7, SCN10A for Nav1.8, SCN11A for Nav1.9. Again, the beta subunit is represented by the Na_v_β signal where it is distinguished by the SCN2B gene for Na_v_β2, SCN3B for Na_v_3β, and SCN4B for Na_v_β4 [95]. At present, various experiments have shown that Navα and Na_v_β subunits are present in the case of non-excitable cancer tissue, such as breast, colon, lung, prostate, and colon cancers. On the other hand, in the case of non-cognate cancerous tissue, the alpha and beta subunits are not deeply evident [96]. In Table 1, the role of each subunit in cancer is described. Now, we will discuss how omega-3 PUFAs prevent cancer by targeting this voltage-gated sodium channel. Omega-3 PUFAs modulate the ion channel by exhibiting anti-invasive and antimetastatic properties. Preliminary tests have shown that omega-3 PUFAs combine directly with the Nav1.5 channel protein to control cancer by reducing its deepest latent state [97]. Later, in the case of human breast cancer cells, the application of DHA at a concentration of 0.5–10 µm has been shown to reduce the expression of the SCN5A gene by binding to the DHA lipid-sensitive nuclear receptor, thereby reducing the latent state of Na_v_1.5. The downstream protagonist NHE-1 helps in cancer prevention by reducing activation and hydrogen ion efflux by reducing extracellular matrix proteolytic activity, which is shown in Figure 5 [98].

## 9. Translational Impact of PUFAs

Omega-3 PUFAs are easily assimilated into the lipid layer of the cell membrane, especially for tumor cells, because these types of fatty acids are amphiphilic. Various experiments have shown that omega-3 PUFAs, in addition to FDA-approved anticancer drugs, also increase the desired efficacy of anticancer drugs and modulate biological membranes [99,100,101,102]. DHA combination sensitized colon cancer cells tonon steroidal anti-inflammatory drug (sulindac sulfide) induced apoptosis, leading to enhanced growth suppression of human colon cancer xenografts. PUFAs have also enhanced the susceptibility of human colorectal cancer cells combined with 5-fluorouracil for mammary carcinoma in addition with celecoxib, tamoxifen in breast cancer, cisplatin in lung cancer, gemcitabine in breast and liver cancers, doxorubicin, vincristine, and fludarabine in leukemia. A total of 550 clinical trials under the investigation of the government suggested that the effect of PUFAs is highly impactful on various chronic diseases in humans [103]. Various investigation results indicated that EPA at 2 g/d for 3 mo also reduces colonic crypt cell proliferation and increases apoptosis in normal colonic mucosa [104]. Alternatively, as part of a parenteral nutrition plan, omega-3 PUFAs can be given as intravenous lipid emulsions. In patients recovering from surgical removal of gastric tumors, omega-3 PUFA emulsion-based parenteral feeding lowers inflammatory response and the rate of inflammatory sequelae [105]. In patients given 3 g of omega-3 PUFA daily for 7 days before surgery for colorectal cancer, EPA was rapidly absorbed into the colonic mucosa and the colonic muscle layer, supporting assertions about the therapeutic benefits of omega-3 PUFA. More clinical research is needed to determine the effect of EPA and/or DHA dose and duration on critical molecular targets, such as cell plasma [106]. Some of the formulations are described in Table 2.

## 10. Conclusions and Future Perspectives

Nowadays, advancement in lipid research plays a vital role in understanding the importance of plasma membrane composition and its structure in chronic diseases such as cancer. Here, we have summarized the role of omega-3 PUFAs in membrane channel integrity and breast cancer prevention. Since membrane lipid composition influences cellular function, future research should consider the role of omega-3 PUFAs in the modulation of membrane integrity and controlling different membrane pumps to alter drug availability. In the future, research based on lipids will provide new techniques and approaches to answer various questions that remain unanswered about the benefits of omega-3 PUFAs.

## Figures and Tables

**Table 1:** Role of various sodium channel subunits in different types of cancer.

Name of Sodium Channel	Type of Cancers	Expression	Mechanism
Nav1.5	Breast	Up-regulated mRNA, and protein	Increase invasion by increasing Src activity and allosteric activation of NHE-1
Nav1.5	Colorectal	Up-regulated mRNA, and protein	Increase invasion by the regulation of transcriptional pathway Pka/Erk
Nav1.5	Ovarian	Up-regulated mRNA, and protein	Increase migration, invasion, and proliferation by increasing the window currently
Nav1.6	Cervix	Up-regulated mRNA, and protein	Increased invasion and boosted activity of MMP2 and NHE-1
Nav1.7	Prostate	Up-regulated mRNA, and protein	Cell motility increased via galvanotaxis
Nav1.7	Lung	Up-regulated mRNA, and protein	Increased invasion and dysregulation of sodium homeostasis, an increase in sodium ion, and depolarization of cell membrane
Navβ1	Breast	Down-regulated mRNA, protein	Increased invasion by decreasing cell adhesion and facilitating cell migration
Navβ1	Lung	Down-regulated mRNA, protein	Increased invasion by decreasing cell adhesion and facilitating cell migration
Navβ2	Prostate	Up-regulated mRNA, and protein	Increased invasion by promotion of bipolar cell morphology enhanced cell adhesion
Navβ3	Bone	Up-regulated mRNA, and protein	Increased apoptosis by increasing the p53 dependent apoptotic pathway
Navβ4	Breast	Down-regulated mRNA, protein	Increased invasion by enhancing RhoA activity

**Table 2:** Contribution of different omega-3 PUFAs for the prevention of different cancer.

Name of PUFA	Type of Cancer	Cell Line and Animal Study	Effective Dose
Eicosapentaenoic acid	B lymphocyte (lymphoblast)	U 266	50 μM
Docosahexaenoic acid	B lymphocyte (lymphoblast)	U 266	100 μM
Eicosapentaenoic acid	Plasma cell leukemia	L363	50 μM
Docosahexaenoic acid	Plasma cell leukemia	L363	100 μM
Arachidonic acid	Prostate cancer	PC3	50 μM
Eicosapentaenoic acid	Prostate cancer	PC3	1 μM
Docosahexaenoic acid	Breast cancer	MDA-MB-231	20 μM
Docosahexaenoic acid	Non-small cell lung cancer	A459	25 μM
Eicosapentaenoic acid	Colon cancer	CR HT-29	20 μmol/L

## Data Availability

Not applicable.
